# Eotaxin-1 (CCL11)

**DOI:** 10.3389/fimmu.2015.00084

**Published:** 2015-02-24

**Authors:** Timothy John Williams

**Affiliations:** ^1^Airway Disease Section, National Heart & Lung Institute, Faculty of Medicine, SAF Building, Imperial College London, London, UK

**Keywords:** eosinophils, eotaxin, chemokines, asthma, allergy

The eosinophil was first named by the brilliant German scientist Paul Ehrlich in 1879, while he was experimenting with aniline dyes to stain blood cells and tissues. He also discovered neutrophils, basophils, and mast cells. The highly basic proteins in cytosolic granules of a small subpopulation of cells in human blood stained vivid pink with the acid dye eosin (from the Greek “eos” meaning dawn), hence “eosinophils.” He subsequently observed high numbers of these cells in the sputum of asthmatic patients and recognized the close relationship between eosinophilia and the severity of asthma. Pertinent to our story was his proposition that a “material which attracts eosinophils” exists. Further, he postulated that eosinophils and neutrophils possess different “chemotactic irritability” and that eosinophils only migrate to sites where a “specific stimulating substance” is present (1).

This could have been the inspiration behind the Eotaxin project but, in truth, its origins were more prosaic. To provide a brief background, my Ph.D. project on mechanisms of inflammation involved the measurement of microvascular plasma protein leakage in rabbit and guinea pig skin using ^125^I-albumin as a marker. This led to an investigation of endogenous mediators that increase the permeability of venules *in vivo* using intradermal zymosan as the inflammatory stimulus. Alternatively, zymosan was administered intraperitoneally in rabbits and the skin system was used as an *in vivo* bioassay for peritoneal exudates collected at intervals. The major finding from all these studies was that the principle permeability-increasing mediator was extravascularly generated C5a. Further, C5a-induced leakage was dependent on a rapid interaction between neutrophils and venular endothelial cells, as evidenced by neutrophil depletion experiments (2): (followed up recently in *J Exp Med*, 2014). We then began experiments with ^111^In-neutrophil trafficking *in vivo*, and the purification and identification of C5a brought us into contact with an expert protein sequencing group in London. In a paper published in 1986, we noted that there was a small amount of permeability-increasing activity, other than C5a, in 2 h zymosan-induced peritoneal exudates. Some time later, we assayed 6 h exudates in the skin in the presence of a C5a neutralizing antibody and identified two potent activities. Purification using HPLC, followed by microsequencing, revealed that these were the rabbit equivalents of IL-8 (CXCL8) and MGSA (CXCL1); results published in 1990 and 1991. Thus, at this stage, our journey had taken us from an interest in the barrier function of the venular endothelium, to the complement system and neutrophils, and then on to chemokines.

By this time, I had moved to the National Heart & Lung Institute in West London to take up a professorial chair funded by a charity, the National Asthma Campaign, later renamed Asthma UK. We seemed to be on another planet; clearly, the world of asthma was orbiting around the eosinophil. There was little interest in the neutrophil (although eventually this changed with a growing emphasis on the heterogeneity of the disease, some asthma subtypes being clearly neutrophilic). To redress the balance, I introduced Lucia Faccioli, a visitor from Brazil, to eosinophil expert Redwan Moqbel in the Institute and we developed a method to measure ^111^In-eosinophil accumulation in guinea pig skin *in vivo*. I later recruited David Griffiths-Johnson who had specialized in lung lavage of allergen-challenged sensitized guinea pigs. The plan to combine the two techniques as an *in vivo* generating and *in vivo* bioassay system to identify endogenous eosinophil chemoattractants was submitted to the asthma charity as a project grant, but sadly this was rejected with not unreasonable reservations about feasibility. Despite this, we continued using funds raised for another project. After several “false dawns,” the pursuit proved successful and in 1992 we were regularly detecting activity in lung lavage fluid, indicated by a strong ^111^In-eosinophil signal in bioassay skin samples. Unfortunately, at this point, we had lost our biochemist, Peter Jose, who had developed the methodology for the purification of rabbit IL-8 and MGSA. Peter had abandoned the hunt for the elusive eosinophil chemoattractant and moved out of science to a rural retreat in Marmande in France. I flew to France clutching the new data and met Peter who seemed more interested in the ripening of his strawberry crop, but was persuaded to return to London to take on the challenge. The lavage fluid was put through a series of HPLC purification stages and within a relatively short time Peter had purified the protein for microsequencing. Within 2 weeks, the sequencing group had an N-terminal sequence of a novel CC chemokine. Soon, they had sequenced peptide proteolytic fragments of the protein and had assembled the full 73-aa sequence. We called this protein “Eotaxin” (condensed from “eosinophil chemotaxin”). We submitted a manuscript to *Nature* and were pleased with the positive reports that came back from two of the referees, which betrayed a North American flavor (“flavor”). The third, more critical, referee appeared to be from the “United” Kingdom and stated that the molecule had not been cloned and that chemotaxis had not been demonstrated *in vitro*. These statements were true, but seemed to miss the point. There was considerable interest in academia and industry in the eosinophil as a therapeutic target in asthma. Activated eosinophils release their highly basic, tissue-damaging proteins, and a range of mediators that can exacerbate lung inflammation. Despite the interest, cloning had not revealed the Eotaxin sequence. We had tried eosinophil chemotaxis *in vitro* as an assay, but the cells were confused by a whole gamut of non-specific stimulants in lavage fluid. In contrast, the *in vivo* skin assay excelled in picking up the chemoattractant “needle” in a “haystack” of irrelevant molecules. After two more unsuccessful attempts at submission to *Nature* with more data, the paper was redrafted and appeared, considerably delayed, in the *Journal of Experimental Medicine* in March 1994 (3).

In early 1994, I received a phone call from Tim Springer inviting me to give a talk at Harvard. After the talk, Tim remarked that Henry Dale (who deduced that histamine was released *in vitro* from tissues of allergen-sensitized guinea pigs in the early 1900s) would have appreciated our approach. I took this as a compliment but, on reflection, this more resembled understandable sarcasm at our “retro” methodology. However, one advantage with our approach was that we could immediately set our molecule in a disease context and give it a rational name. With the encouragement of Craig Gerard, Tim invited me to join the scientific advisory board of LeukoSite Inc., the company that he had recently founded with Eugene Butcher. The Eotaxin patents were licensed to the Company and a successful relationship ensued. Based on the protein sequence (Figure 1) we cloned guinea pig Eotaxin in a previously established collaboration with Christine Power at GSK in Geneva. Marc Rothenberg at Harvard then used the guinea pig protein sequence to clone mouse Eotaxin and embarked on a series of experiments with allergy models in Eotaxin knockout mice (4) followed by important papers demonstrating a role for Eotaxin, particularly in diseases of the GI tract. Paul Ponath and colleagues at LeukoSite cloned human Eotaxin (5) and its receptor CCR3. There were several publications on these sequences around this time. Subsequent discoveries in other laboratories published in 1997 and 1999 revealed two more Eotaxins signaling through CCR3, Eotaxin-2 (CCL24), and -3 (CCL26), with low sequence similarity to the renamed Eotaxin-1, designated CCL11 of the CC chemokine family. We used immunoassays to investigate the role of Eotaxin-1 in the guinea pig allergy model (6), and Alison Humbles then moved to Craig Gerard’s laboratory to investigate mouse models using CCR3 knockout mice. Many groups, including ours, published papers showing the expression of Eotaxin in human asthma.

**Figure 1:**
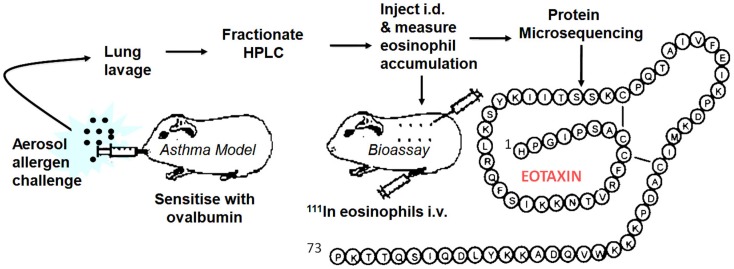
**Generation, bioassay, purification, and sequencing of Eotaxin-1**.

A recurring question from eosinophil experts concerned the relationship between Eotaxin and the cytokine, IL-5, which was reported to be an eosinophil chemoattractant. Contrary to the reports, we found that IL-5 did not induce eosinophil accumulation when injected into guinea pig skin. However, intravenous IL-5 induced the release of eosinophils from the bone marrow reserve and the dramatic increase in circulating cells in the blood markedly enhanced eosinophil recruitment induced by intradermal Eotaxin-1 (7). We subsequently developed an *in situ* bone marrow perfusion system to study mechanisms of eosinophil release in detail.

As at LeukoSite, many companies developed CCR3 antagonists. Ian Sabroe in my laboratory devised a technique to measure responses of human eosinophils to chemokines (the “GAFS” Shape Change Assay, now widely used in academia and industry) and discovered a subpopulation of donors whose cells responded to CCR3 and CCR1 agonists. This led to the first publication on a CCR3 antagonist (8), one that could also antagonize CCR1. There have been clinical trials of CCR3 antagonists in asthma patients but, as yet, no drug has reached the market. Cambridge Antibody Technology (now MedImmune) produced a potent therapeutic antibody (CAT-213, iCo-008, Bertilimumab) that neutralizes Eotaxin-1. This antibody, tested initially in allergic rhinitis, has been licensed to iCo Therapeutics and to Immune Pharmaceuticals, for testing in further clinical trials. As well as being evaluated as a therapeutic target in asthma and in allergic diseases in general, Eotaxin-1 is used as a biomarker in clinical trials. There is also interest in diseases of the GI tract where Rothenberg’s work has been particularly influential, with trials planned in ulcerative colitis and Crohn’s disease. In addition, Eotaxin-1 is implicated in diseases, such as atherosclerosis, apparently independently of its action on eosinophils. Interestingly, as published in *Nature* in 2009, CCR3 is expressed on endothelial cells in vessel overgrowth of the macula in age-related macular degeneration (AMD) and locally produced Eotaxins are thought to mediate angiogenesis in this condition [see Ref. (9)]. Thus, Bertilimumab is being considered for the treatment of AMD and other eye diseases. There is also evidence, from cross-circulation studies between old and young mice that circulating Eotaxin-1 rises during aging and this suppresses neurogenesis and cognitive function, as published in *Nature* in 2011 [see Ref. (9)], raising possibilities for future therapy in dementia.

Thus, from humble origins, the work on Eotaxin-1 has raised tantalizing opportunities for therapy ranging across several diseases. These possibilities have not yet translated into effective therapy, but we are not alone in this in the chemokine field (9).

## Conflict of Interest Statement

The author is a named “inventor” on a patent of the Eotaxin-1 molecule.

## References

[1] Gleich GJ Historical overview and perspective on the role of the eosinophil in health and disease. In: Lee JJ Rosenberg HF, editors. Eosinophils in Health and Disease. Waltham: Academic Press (2013). p. 1–11 10.1016/B978-0-12-394385-9.00001-8

[2] Wedmore CV Williams TJ. Control of vascular permeability by polymorphonuclear leukocytes in inflammation. Nature (1981) 289:646–50. 10.1038/289646a0 7464931

[3] Jose PJ Griffiths-Johnson DA Collins PD Walsh DT Moqbel R Totty NF Eotaxin: a potent eosinophil chemoattractant cytokine detected in a guinea pig model of allergic airways inflammation. J Exp Med (1994) 179:881–7. 10.1084/jem.179.3.881 7509365 PMC2191401

[4] Rothenberg ME MacLean JA Pearlman E Luster AD Leder P. Targeted disruption of the chemokine eotaxin partially reduces antigen-induced tissue eosinophilia. J Exp Med (1997) 185:785–90. 10.1084/jem.185.4.785 9034156 PMC2196140

[5] Ponath PD Qin S Ringler DJ Clark-Lewis I Wang J Kassam N Cloning of the human eosinophil chemoattractant, eotaxin. Expression, receptor binding, and functional properties suggest a mechanism for the selective recruitment of eosinophils. J Clin Invest (1996) 97:604–12. 10.1172/JCI118456 8609214 PMC507095

[6] Humbles AA Conroy DM Marleau S Rankin SM Palframan RT Proudfoot AE Kinetics of eotaxin generation and its relationship to eosinophil accumulation in allergic airways disease: analysis in a guinea pig model *in vivo*. J Exp Med (1997) 186:601–12. 10.1084/jem.186.4.601 9254658 PMC2199038

[7] Collins PD Marleau S Griffiths-Johnson DA Jose PJ Williams TJ. Cooperation between interleukin-5 and the chemokine eotaxin to induce eosinophil accumulation *in vivo*. J Exp Med (1995) 182:1169–74. 10.1084/jem.182.4.1169 7561691 PMC2192289

[8] Sabroe I Peck MJ Van Keulen BJ Jorritsma A Simmons G Clapham PR A small molecule antagonist of chemokine receptors CCR1 and CCR3. Potent inhibition of eosinophil function and CCR3-mediated HIV-1 entry. J Biol Chem (2000) 275:25985–92. 10.1074/jbc.M908864199 10854442

[9] Solari R Pease JE Begg M. Chemokine receptors as therapeutic targets: why aren’t there more drugs? Eur J Pharmacol (2015) 746:363–7. 10.1016/j.ejphar.2014.06.060 25016087

